# The PINK1-VCP-p47 pathway protects against tau-induced dendritic shrinkage in cortical neurons through interaction with STIP1

**DOI:** 10.1016/j.nbd.2026.107592

**Published:** 2026-08-29

**Authors:** Sukanya Banerjee, Kent Z.Q. Wang, Michael R. DeChellis-Marks, Madeleine R. Ross, Nadine H. Oury, Charleen T. Chu

**Affiliations:** a Department of Pathology, Division of Neuropathology, University of Pittsburgh School of Medicine, Pittsburgh, PA, USA; b Department of Neurobiology, LiveLikeLou Center for ALS Research, University of Pittsburgh School of Medicine, Pittsburgh, PA, USA; c Medical Scientist Training Program, University of Pittsburgh School of Medicine, Department of Neurobiology and Pittsburgh Center for Pain Research, University of Pittsburgh, Pittsburgh, PA, USA

**Keywords:** Microtubule-associated protein tau, Dendritic simplification, p47 phosphorylation, PINK1, Neuroprotection, STIP1, Tauopathy

## Abstract

Microtubule-associated protein tau (MAPT) contributes to the pathogenesis of Alzheimer’s disease (AD) and some forms of Frontotemporal Lobar Dementia wherein dendritic simplification is strongly related to cognitive dysfunction. Loss of function mutations in PTEN-induced kinase 1 (PINK1) cause recessive early-onset Parkinson’s disease with dementia, and reduced wild-type PINK1 expression is observed in sporadic AD. We previously found that PINK1 interacts with valosin-containing protein (VCP) and promotes phosphorylation of the VCP cofactor NSFL1C (p47). This study was designed to test the hypothesis that PINK1 rescues tau-induced dendritic simplification through the VCP-p47 pathway, and to define neuroprotective mechanisms downstream of p47 phosphorylation. Mouse primary cortical neurons were transfected with 2N4R-Tau and plasmids encoding components of the PINK1-VCP-p47 signaling pathway and the impact on tau-mediated dendritic injury was assessed. Immunoprecipitation-mass spectrometry, immunoblotting, RNAi and Sholl analysis were performed to define downstream mechanisms of neuroprotection. Tau-induced dendritic shortening and simplification was fully rescued by co-expression of PINK1, VCP, p47 or the p47-S176D phosphomimic (p47D), but not by the non-phosphorylatable p47-S176A (p47A) variant. Pathway analysis showed enhanced interactions of p47D with proteins in the Rho-GTPase pathway. Immunoprecipitation confirmed that STIP1 interacts with p47D to a significantly higher degree than p47A. Overexpression of STIP1 rescued tau-mediated dendritic simplification. Moreover, STIP1 siRNA impaired the neuroprotective effects of both PINK1 and p47D, indicating an essential role in the PINK1-p47D neuroprotection pathway. The current study highlights a novel PINK1-(phospho)p47-STIP1 signaling axis that promotes dendritic complexity and protects against tau-induced neurodegeneration in cortical neurons.

## 1. Introduction

Tauopathies are neurodegenerative disorders characterized by abnormal aggregates of the *MAPT* gene product microtubule-associated protein tau in the brain (Gotz et al., 2019; Zhang et al., 2022). Tau proteins act to stabilize microtubules, maintaining the structural integrity of neurons and regulating signal transmission between neurons and axonal transport (Wang and Mandelkow, 2016). Tau pathology is frequently composed of hyperphosphorylated tau (He et al., 2018), which disassembles from microtubules and aggregates to form neurofibrillary tangles (NFTs) (Zhang et al., 2018). Human tau is classified as 3R tau or 4R tau isoforms based on the number of microtubule-binding domains present (Gotz et al., 2019; Xia et al., 2019). Overproduction of 4R tau is pathogenic in multiple neurodegenerative diseases (Zhang et al., 2018; Peck et al., 2011). While NFTs of Alzheimer’s disease (AD) patients contain both 3R and 4R tau isoforms (Arai et al., 2003), pathological deposits of 4R tau in neurons and microglia are characteristic of several frontotemporal lobal dementias (FTLD) (Flament et al., 1991). Tau pathologically accumulates in the somatodendritic compartment in cultured neurons and neurons of AD mice (Zempel et al., 2017), accompanied by loss of dendritic spines (Zempel et al., 2017; Ittner and Ittner, 2018). Loss of dendritic arborization is a characteristic feature shared among patients with various forms of dementia (Terry et al., 1991; Lacor et al., 2007).

Autosomal recessive *PINK1* mutations cause early-onset parkinsonism (Ricciardi et al., 2014), and reduced wild-type PTEN-induced kinase 1 (PINK1) expression is observed in sporadic AD (Du et al., 2017). PINK1 has been heavily implicated in regulating mitophagy, the autophagy–lysosome pathway, the ubiquitin–proteasome system, neurite outgrowth and neuron survival, inflammation, tumor suppression, and apoptosis (Cummins and Gotz, 2018; Michiorri et al., 2010; McLelland et al., 2014; Parganlija et al., 2014; Akabane et al., 2016; Arena and Valente, 2017). PINK1 regulates dendritic arborization (Dagda et al., 2014; Wang et al., 2018), with *pink−/−* neurons exhibiting decreased synaptic input and reduced spine density and maturation (Otero et al., 2022). Elevation of human PINK1 rescues dendritic simplification and dendritic spines in *pink−/−* mice (Otero et al., 2022).

Mature PINK1 is known to interact with valosin-containing protein (VCP/p97) (Wang et al., 2018; Guardia-Laguarta et al., 2019). VCP belongs to the type II AAA+ ATPase family of proteins with many cellular activities, including degradative extraction of ubiquitinated organellar proteins, golgi remodeling, membrane trafficking, cell cycle, and transcriptional regulation (Bento et al., 2018). The interaction of PINK1 and VCP increases dendritic complexity through phosphorylation of the VCP cofactor p47, also known as NSFL1C, in *pink1−/−* mouse neurons (Wang et al., 2018). Overexpression of a p47-S176D phosphomimic (p47D) can substitute for PINK1 in rescuing dendrite simplification in *pink1−/−* mouse neurons (Wang et al., 2018).

The present study aims to explore the neuroprotective potential of this novel PINK1-VCP-p47 signaling pathway against tau-mediated neuron injury. Using mouse primary cortical neurons, we observed that 2N4R-tau caused dendritic simplification, which was rescued by overexpressed PINK1 or VCP. Elevation of the VCP co-factor p47 and the p47D phosphomimic were also neuroprotective against tau-induced dendritic simplification. Using unbiased methods, we identified and validated a novel mediator acting downstream of p47D, Stress-induced phosphoprotein 1 (STIP1). Knockdown studies confirm STIP1 is an essential mediator for PINK1- and p47-mediated neuroprotection against dendritic simplification.

## 2. Methods

### 2.1. Primary cortical neuron culture

Principles of laboratory animal care and handling were followed (NIH publication No. 86–23, rev. 1985), and all procedures for primary culture were approved by the University of Pittsburgh Institutional Animal Care and Use Committee. Primary cortical neurons were isolated from E15–16 C57BL/6 mice. Isolated cortices from male and female pups were pooled and incubated for 4–6 min with 200 units papain (Worthington Biochemical) dissolved in dissociation media (10 mM MgCl_2_, 1 mM kynurenic acid,10 mM HEPES in 1× Hank’s Balanced Salt Solution (w/o Mg^++^, Ca^++^) and activated by L-cysteine. After removal of papain, cortices were washed with 0.1 mg/ml ovomucoid protease inhibitor (Worthington Biochemical) in dissociation media, then transferred to neurobasal media (Gibco) supplemented with 2% B27 (Gibco), 1 mm l-glutamine (Invitrogen), and 10% FBS. Cortices were then isolated to single cell suspension by gentle, manual trituration using a 1000 μl pipet. Isolated cortical neurons were plated on prewashed poly-ornithine coated 24 well plates.

### 2.2. Plasmid transfection

Mouse primary cortical neurons (wild type) were cultured and transfected at 7 d *in-vitro* (DIV7) with expression plasmids encoding Venus, a point mutant of GFP that shows greater brightness and reduced sensitivity to pH, and 2N4R-Tau (hereafter referred to as Tau) using Lipofectamine 2000. Total plasmid DNA was kept at 1 μg per well for all transfection conditions using pcDNA control vector. Some cultures were rescued by co-transfection with human PINK1, VCP, p47, p47(S176D; p47D), p47(S176A; p47A) (Wang et al., 2018) or STIP1. Neurons were fixed and immunofluorescence was performed 7d after transfection (at DIV14) to study neuronal morphology.

### 2.3. Immunoprecipitation-mass spectrometry (IP-MS)

For IP-MS, 1 × 10^7^ SH-SY5Y cells were transfected with 20 μg plasmid encoding GFP (control), MYC-p47A or MYC-p47D using Lipofectamine 2000 (Invitrogen) for 48 h. Immunoprecipitation was performed using a magnetic c-myc isolation kit (Miltenyi Biotec, Cat#130–091-123). The eluted control, MYC-p47A or -p47D bound proteins were electrophoresed into a 10% SDS polyacrylamide gel. After the Coomassie blue dye front was completely migrated into the gel, the entire blue region was excised, washed in buffer containing 40% methanol and 7.5% acetic acid, washed twice in 50% methanol/5% acetic acid for 60 min, washed with acetonitrile for 5 min, then dried and suspended in 75 μl 50 mM ammonium bicarbonate (with 5 mg/ml DTT) for 30 min at RT. The DTT-containing ammonium bicarbonate was aspirated and the gel pieces were suspended in 50 mM ammonium bicarbonate containing 15 mg/ml iodoacetamide (75 μl) and incubated for 30 min in the dark at RT. The gel pieces were alternately incubated with 50 mM ammonium bicarbonate and acetonitrile, twice, respectively, for 5 min each (total of four incubations) and then completely dried. Dried gel slices were rehydrated with 25 μl of 50 mM ammonium bicarbonate. Then 75 μl of sequencing grade-modified trypsin (Promega, Madison WI) prepared at 10 μg/ml in 50 mM ammonium bicarbonate was added and driven into the gel pieces for 15 min at 37 °C. Finally, 25 μl 50 mM ammonium bicarbonate was added to the trypsin mix and digestion was allowed to take place overnight. The peptides were extracted from the polyacrylamide with 50 μl 50% acetonitrile and 5% formic acid three times. A final extraction with 50 μl of acetonitrile was performed. The extracted pool was completely dried in a vacufuge and peptides were resuspended in 20 μl of 50 mM acetic acid.

For target validation, HEK293 cells were transfected using GFP, Myc-GFP, p47A, or p47D using Lipofectamine 3000. Cells were lysed after 72 h of transfection. Co-immunoprecipitation was performed using the mMacs c-myc isolation kit (#130–091-123) according to the manufacturer’s protocol. Samples were stored at −80 °C and input and pulldown samples probed by immunoblot.

#### 2.3.1. Orbitrap fusion

Capillary-liquid chromatography-nanospray tandem mass spectrometry (Capillary-LC/MS/MS) protein identification was performed on a Thermo Scientific orbitrap Fusion mass spectrometer equipped with an EASY-Spray^™^ Sources operated in positive ion mode. Samples (6.4 μl) were separated on an easy spray nano column (Pepmap^™^ RSLC, C18 3 μ 100 A, 75 μm X150mm Thermo Scientific) using a 2D RSLC HPLC system from Thermo Scientific. Each sample was injected into the μ-Precolumn Cartridge (Thermo Scientific) and desalted with 0.1% formic acid in water for 5 min. The injector port was then switched to inject, and the peptides were eluted off the trap onto the column. Mobile phase A was 0.1% formic acid in water and acetonitrile (with 0.1% formic acid) was used as mobile phase B. Flow rate was set at 300 nl/min. For in gel digestion samples, mobile phase B was increased from 2% to 5% in 5 min and then increased from 5 to 16% in 40 min and again from 16%–25% in 10 min and then from 25 to 32% in another 10 min. Mobile phase B was increased again to 90% in 1 min and then kept at 90% for another 2 min before being brought back quickly to 2% in 1 min. The column was equilibrated at 2% of mobile phase B (or 98% A) for 12 min before the next sample injection.

MS/MS data was acquired with a spray voltage of 1.6 KV and a capillary temperature of 305 °C. The scan sequence of the mass spectrometer was based on the preview mode data dependent TopSpeed^™^ method: the analysis was programmed for a full scan recorded between *m/z* 375–1500 and a MS/MS scan to generate product ion spectra to determine amino acid sequence in consecutive scans starting from the most abundant peaks in the spectrum in the next 3 s. To achieve high mass accuracy MS determination, the full scan was performed at FT mode and the resolution was set at 120,000 with internal mass calibration. The AGC Target ion number for FT full scan was set at 4 × 10^5^ ions, maximum ion injection time was set at 50 ms and micro scan number was set at 1. MSn was performed using HCD in ion trap mode to ensure the highest signal intensity of MSn spectra. The HCD collision energy was set at 32%. The AGC Target ion number for ion trap MSn scan was set at 3.0E4 ions, maximum ion injection time was set at 35 ms and micro scan number was set at 1. Dynamic exclusion is enabled with a repeat count of 1 within 60s and a low mass width and high mass width of 10 ppm.

#### 2.3.2. Data analysis

Sequence information from the MS/MS data was processed by converting the .raw files into a merged file (.mgf) using MSConvert (ProteoWizard). Isotope distributions for the precursor ions of the MS/MS spectra were deconvoluted to obtain the charge states and monoisotopic *m/z* values of the precursor ions during the data conversion. The resulting mgf files were searched using Mascot Daemon by Matrix Science version 2.5.1 (Boston, MA) against Uniprot database 2018_10. The mass accuracy of the precursor ions was set to 10 ppm, accidental pick of 1 ^13^C peaks was also included into the search. The fragment mass tolerance was set to 0.5 Da. Carbamidomethylation (Cys) is used as a fixed modification and considered variable modifications were oxidation (Met) and deamidation (N and Q). Four missed cleavages for the enzyme were permitted. A decoy database was also searched to determine the false discovery rate (FDR) and peptides were filtered according at 1% FDR. Proteins identified with at least two unique peptides were considered as reliable identification. Any modified peptides are manually checked for validation. MS data was analyzed using Scaffold (Proteome Software, Inc., version 5.1.2). MS/MS spectra were searched against the Uniprot human database release using MASCOT (Brosch et al., 2009), version 2.6.2; missed cleavages = 4; fragment mass tolerance = 0.50 Da; parent mass tolerance = 5.0 PPM; dynamic modifications: Deamidated / + 0.98402 Da, Oxidation / + 15.995 Da; static modifications: Carbamidomethyl / +57.021 Da). Each sample peptide probability model was developed using PeptideProphet (Ma et al., 2012) with delta mass corrections ranging from +2 to +7. Peptide spectral matches were filtered using a false discovery rate (FDR) of 1. Quality control check revealed an absence of p47 peptides in the D3 replicate of p47D immunoprecipitation, which was excluded from further analysis. Quantitative protein values from A1, A2, A3, D1, D2, D4 biological replicates were normalized to total spectra, and the values from GFP-control subtracted. Quantitative protein analysis was limited to proteins with values greater than zero in at least 66% of samples per group. Differential protein levels were measured as log-2 ratios of S176D to S176A and statistically measured using a two-tailed *t*-test. Ingenuity Pathway Analysis (IPA, Qiagen) was used to generate canonical pathway enrichment profiles.

#### 2.3.3. RNAi transfection

Mouse primary cortical neurons cultured and transfected at DIV7 were treated with 25 nM nontargeting siCtrl (sictrl; Sigma) and siRNA targeting p47 (sip47; Dharmacon) or STIP (siSTIP1; Dharmacon) using Lipofectamine 2000 at DIV7, DIV9 and DIV12. A subset of neurons was lysed on DIV14 to verify successful target knockdown. The remaining neurons were fixed for morphological analysis.

### 2.4. Immunostaining and neuronal morphometry analysis

Mouse primary cortical neurons were fixed in 4% paraformaldehyde (2% final; Sigma-Aldrich) at room temperature for 20 min and washed with 1× PBS, followed by methanol for 5 min. Fixed cells were washed and permeabilized with 0.1% Triton X-100 in PBS for 20 min followed by PBS-washes. After treatment with Superblock Buffer (Thermo Scientific) for 1 h at room temperature, cells were stained with primary antibodies (Additional file 7: Table S1) at 4 °C overnight. After PBS-washes, the appropriate secondary antibodies (Additional file 8: Table S2) were added for 1 h at room temperature. The cells were washed, stained with Hoechst (1:500 in 1× PBS) for 5 min, and washed. Single plane images were captured using an Olympus IX83 microscope with a DP80 camera (40× oil-immersion objective, numerical aperture 1.3). Sholl analysis of pyramidal neurons was performed using National Institutes of Health ImageJ software, supplemented with the SNT plug-in, as previously described (Otero et al., 2022). Blinded Sholl image analysis was performed by individuals previously trained using a standardized dataset, achieving Pearson r values of >0.8 relative to S.B.

### 2.5. Western blotting

Total protein was extracted from HEK293 cells using lysis buffer (HEPES, NaCl, EDTA. Glycerol, 1% triton X, pH 7.5) in the presence of protease and phosphatase inhibitors (E64, NaPP, PMSF and sodium orthovanadate). Lysates were centrifuged, and the supernatant (soluble) was collected and quantified by Bradford Assay. The pellets were washed two times with lysis buffer, and the wash buffer was discarded. Then, the pellets (insoluble) were dissolved in SDS-PAGE loading buffer. The soluble and insoluble protein fractions were loaded onto SDS-PAGE gels separately and electroblotted using a wet transfer system. Membrane was blocked with 5% skim milk in 1× PBST at room temperature for 1 h. The membrane was then incubated with the primary antibody (Additional file 7: Table S1) at 4 °C overnight. The next day, the membrane was incubated with secondary antibody (Additional file 8: Table S2) for 1 h at room temperature, then developed with a chemiluminescent substrate. Western blot analysis was performed using ImageStudio software.

### 2.6. Statistical analysis

Sholl curves were analyzed by repeated-measures ANOVA with Tukey multiple comparison post-hoc test using GraphPad Prism 10 software (version 10.1.0). For tau densitometry, each lane was normalized to the total intensity of all lanes on that blot (blot-wide sum normalization); triplicate lanes were averaged for each independent experiment. Two-group data were analyzed using unpaired 2-tailed *t*-test. One-way ANOVA with Tukey’s multiple comparison post-hoc test or Brown–Forsythe ANOVA with Dunnett’s T3 multiple comparisons test was used for multiple group comparison for equal and unequal variances, respectively, using GraphPad Prism 10 software. A *p* value *<*0.05 was considered significant. Error bars in the scatter plots depict mean +/− S.D.

## 3. Results

### 3.1. 2N4R-Tau causes dendritic simplification and PINK1 elevation protects against tau-mediated dendrite simplification

Primary mouse cortical neurons were co-transfected at DIV7 with Venus, to visualize neuronal processes, and His-tagged Tau or pcDNA vector. Transfected neurons were fixed at DIV14, and stained for His and GFP to verify co-expression (Fig. 1A–1C). Sholl analysis reveals that Tau elicited significant dendritic simplification (Fig. 1D), with significantly reduced Sholl AUC values (Fig. 1E). The summated dendrite length was also significantly reduced in tau-expressing neurons (Fig. 1F). These findings corroborate studies in the literature showing that increases in wild type 2N4R-tau, as observed in several sporadic human neurodegenerative diseases, is sufficient to cause neuronal injury (Adams et al., 2009; Buchholz and Zempel, 2024; Roy et al., 2025; Lo and Sachs, 2020).

Co-transfection of tau with PINK1 was performed to determine the ability of PINK1 to protect against tau (Fig. 1C). We observed rescue of each parameter of tau-induced dendritic simplification by PINK1 (Fig. 1D–1F).

To determine if changes in tau expression were linked to neuroprotection by PINK1, we performed western blot analysis using antibodies to phospho-tau species that are present in post-mortem neurofibrillary tangles (PHF1, phospho-tau (Ser 404)) and antibodies that recognize total tau (Tau-5, total Tau) in HEK293 cells. PINK1 expression did not elicit significant changes in phosphorylated or total tau expression in the soluble fraction (Fig. 1G–1H; See Additional file 1: Fig. S1 A–C for densitometric quantification of soluble blots). Moreover, PINK1 elevation did not change phosphorylated and total tau levels in the insoluble fraction (Fig. 1I) (See Additional file 1: Fig. S1D for densitometric quantification of insoluble blots). Thus, PINK1-mediated neuroprotection is not linked to significant alterations in soluble or insoluble tau levels.

### 3.2. VCP protects against tau-induced dendritic simplification and its cofactor p47 is essential for PINK1 mediated neuroprotection

Similar to PINK1, co-expression of VCP (Fig. 2A–2F) or the VCP cofactor p47 (Fig. 2G–2L) rescued all parameters of dendritic simplification (# intersections, AUC and summated dendrite lengths) induced by tau. Using siRNA targeting p47 (Fig. 3A–3G), we tested the hypothesis that p47 expression is essential for PINK1-mediated neuroprotection. PINK1-mediated rescue was intact in the presence of nontargeting control siRNA (siCtrl). However, upon p47 knockdown, PINK1 no longer rescued dendritic complexity as analyzed by Sholl analyses (Fig. 3E–F) or summated dendrite length (Fig. 3G). These data suggest that PINK1 requires p47 to protect against tau.

### 3.3. Phosphorylation of p47 promotes dendritic complexity

Primary cortical neurons from WT mice were co-transfected with the respective vector control or tau and either non-phosphorylatable p47A or the phosphomimic p47D. Sholl analysis revealed significant rescue of tau-induced injury by p47D but not p47A (Fig. 3H–3I). Likewise summated dendrite length was rescued only by p47D (Fig. 3J). Taken together, these data suggest that phosphorylation of p47 is required for neuroprotection.

### 3.4. IP-MS reveals differentially enriched proteins for p47D including STIP1

PINK1 interacts with VCP to promote phosphorylation of p47 at S176, which has previously been shown to protect against dendritic simplification in *pink1−/−* neurons (Wang et al., 2018). The current data suggests that this pathway also rescues dendritic arborization in the context of tau-mediated dendritic injury. S176 is present in a disordered loop adjacent to the SEP domain of p47 (Fig. 4A). To identify potential mediators downstream of p47 phosphorylation, we utilized IP-MS to identify proteins that differentially interact with the phosphomimic p47D relative to the non-phosphorylatable p47A using three independent biological replicates per group. We identified 1888 proteins and quantified 1256 proteins that were present in at least 66% of both p47A and p47D samples (Fig. 4B). Among these, 34 were nominally enriched in the p47A group (Fig. 4C). Pathway analysis of the p47A enriched and exclusive proteins revealed pathways regulating RNA biology, protein translation and mesodermal signaling (Additional file 2: Fig. S2).

In addition, there was a set of 465 proteins exclusive to the p47D pulldown samples (Fig. 4B). Pathway analysis of these proteins revealed a different set of pathways including mitochondrial translation and the RHO GTPase cycle (Fig. 4D). Given the known role of RHO GTPases in dendritic arborization and spinogenesis (Stankiewicz and Linseman, 2014), we ranked the 26 p47D-interacting proteins in this pathway in order of average spectral abundance after normalizing to the spectral abundance of p47 pulled down in each biological replicate (Table 1). We searched for antibodies for the top 8 targets, and identified commercial antibodies for SPTAN, SCRIB, STIP1, and MYO6. Neither the SPTAN nor the MYO6 antibodies showed specific staining of an appropriately sized band, while SCRIB showed a persistent band in negative control pulldowns. The STIP1 antibody detected appropriately sized bands, with specificity confirmed using RNAi (Fig. 5 insets). Co-immunoprecipitation studies confirmed that significantly more STIP1 is pulled down by p47D than p47A (Fig. 4E–F).

### 3.5. STIP1 is necessary and sufficient for neuroprotection downstream of PINK1 and p47

We used RNAi targeting STIP1 to determine if knockdown of STIP1 would affect the ability of PINK1 to protect against tau (Fig. 5A–5G). In the presence of nontargeting siCtrl, PINK1 rescued each of the Sholl and dendrite length parameters. However, upon STIP1 knockdown, PINK1 no longer rescued dendritic complexity as analyzed by Sholl analyses (Fig. 5E–F) or summated dendrite length (Fig. 5G). These data indicate that STIP1 is necessary for PINK1-mediated neuroprotection.

In parallel experiments, siSTIP1 impaired the ability of p47D to protect against tau-mediated dendrite simplification (Fig. 5H–5J). Finally, elevating STIP1 was itself sufficient to significantly protect against tau-mediated dendritic simplification (Fig. 6).

## 4. Discussion

Dendritic loss, impaired synaptic function and cognitive decline are central features of AD and other tauopathies (Hoover et al., 2010; Zhao et al., 2016). Dendritic simplification is a marker of early injury after exposure to pathogenic species of tau (Sun et al., 2021). Although *in vivo* studies demonstrated that PINK1 overexpression protects against memory deficits in tauopathy mice (Jiang et al., 2021), the underlying mechanism remained unclear. Here, we report that 2N4R-tau elicits dendritic simplification, a measure of neurodegeneration (Grutzendler et al., 2007; Siskova et al., 2014). Tau-induced dendritic simplification could be fully rescued by components of a novel PINK1-VCP-p47 signaling pathway involving p47 phosphorylation. IP-MS data implicate a gain of binding for the p47D phosphomimic to STIP1, an adapter in the Rho GTPase and chaperone pathways. RNAi studies show that STIP1 is essential for neuroprotection downstream of both PINK1 and p47D. Furthermore, elevating levels of STIP1 is sufficient to protect the dendritic arbor from tau-induced simplification.

PINK1 has been extensively studied in the field of mitochondrial quality control (Lizama et al., 2021). Interestingly, enhanced mitophagy inhibits both Aβ and tau pathology in animal models of AD and improves memory performance (Fang et al., 2019; Kingwell, 2019), and ULK1 activators act to reduce tau pathology through induction of autophagy/mitophagy (Pan et al., 2026). However, PINK1 also shows several non-mitochondrial or cytosolic functions (Dagda et al., 2014; Steer et al., 2015). Primary cortical neurons of *pink−/−* mice exhibit decreased dendrite lengths (Dagda et al., 2014; Otero et al., 2022), rescued by introduction of human PINK1. Inhibiting PINK1 degradation acts to elevate endogenous PINK1 levels, conferring protection against MPP+ and 6-OHDA mediated dendrite shortening (Liu et al., 2020). These previous observations align with our current observation of robust neuroprotection against tau-induced dendritic simplification.

Interestingly, overexpression of a form of PINK1 that is tethered to the mitochondrial outer membrane, which is constitutively activated in driving mitophagy, is unable to increase dendritic branching (Dagda et al., 2014). Instead, cytosolic PINK1 interacts with VCP to regulate dendritic branching through p47 (Wang et al., 2018). Cytosolic pools of PINK1 may control dendritic morphology by elevating PKA (Protein Kinase A) activity (Dagda and Das Banerjee, 2015), as PINK1 and PKA interact to modulate mitochondrial trafficking in dendrites (Das Banerjee et al., 2017). A previous study showed that VCP and p47 act together to modulate protein synthesis and dendritic spine formation in neurons (Shih and Hsueh, 2016). Our data further highlight STIP1 as a downstream mediator of the PINK1-VCP-p47 pathway, as p47D requires STIP1 to protect against tau-induced dendritic simplification.

A recent study showed that co-overexpression of PINK1 and human tau in mice over a one month period rescued tau-induced neuron cell death, improved learning and memory tasks, and increased the degradation of both phosphorylated and total tau in hippocampal lysates (Jiang et al., 2021). Thus, we investigated if PINK1 overexpression modulated levels of phosphorylated tau or total tau in the soluble and insoluble compartments of primary neurons. There were no trends to suggest that PINK1 acted to reduce soluble tau levels in our model system; moreover, we did not observe any changes in phosphorylated and total tau levels in the insoluble fraction. One difference is that the prior study utilized a longer period of tau overexpression that was sufficient to cause neuronal cell death (Jiang et al., 2021), whereas our study is restricted to early sublethal phases of injury. Dendritic simplification is an early marker of tau-mediated injury prior to formation of aggregates (Sun et al., 2021). The current data show that PINK1 supports dendritic health and arborization through mechanisms that do not require reductions in tau or phospho-tau levels in a sublethal neuronal injury setting.

Previous studies showed that PINK1 interacts with VCP and promotes phosphorylation of the VCP cofactor NSFL1C/p47 in *pink1−/−* mice (Wang et al., 2018). Expression of the p47D phosphomimic recapitulates the rescue of dendritic complexity by human PINK1 in *pink1−/−* mice (Wang et al., 2018) and significantly reverses dendritic spine loss observed in *pink1−/−* mice (Otero et al., 2022). VCP is also known to show tau-disaggregase activity (Darwich et al., 2020), and VCP overexpression significantly reduces phosphorylated, aggregated species of tau with improvement of tau-induced cognitive decline (Giong et al., 2024). One known limitation of cultured neuron studies is that protein aggregation is not typically observed without additional insults (Amorim et al., 2023; Guo and Lee, 2013). Future studies will be necessary to determine if disaggregating functions of VCP may also contribute to neuroprotection downstream of 2N4R-tau. Interestingly, while VCP itself was able to confer significant protection, the effects were not as robust as observed with PINK1, p47 and STIP1. This has been observed in other systems (Xue et al., 2016; Shah et al., 2025) and may reflect that VCP is involved in multiple other pathways mediated by a large number of other VCP adaptor proteins, many of which compete for the same binding site as p47.

In neurons, VCP and its cofactor p47 cooperate with the endoplasmic reticulum morphology regulator (ALT1), which is known to control ER structure formation and protein synthesis, to regulate dendritic spine formation (Shih and Hsueh, 2016). Mimicking phosphorylation of p47 is sufficient to rescue dendritic branching (Wang et al., 2018) and overall spine densities (Otero et al., 2022) in *pink1−/−* mice. The current data showing that the p47D phosphomimic also rescues dendritic complexity downstream of tau-induced injury supports a general role for this pathway in promoting neuronal arborization.

IP-MS studies were performed to identify potential downstream targets of phosphorylated p47. These data implicated Rho GTPase and mitochondrial translation pathways as potential mediators of neuroprotection downstream of p47. Given the prominent role of Rho GTPases in regulating dendritic arborization, spine formation, growth cones and axon guidance, we validated the enhanced interaction of the p47 phosphomimic with STIP1, a highly ranked component of the Rho GTPase pathway. Impairment of STIP1 function alters Hsp70/Hsp90 expression and activity (Beraldo et al., 2013; Lee et al., 2012; Rohl et al., 2015) and promotes tau accumulation (Ambegaokar and Jackson, 2011). Rescuing chaperone interactions reduces tau or amyloid β toxicity *in vitro* (Lackie et al., 2020; Inda et al., 2020).

Here, we observed that STIP1 overexpression protected against tau-induced dendritic injury. Furthermore, siRNA-mediated knockdown of STIP1 reveals that both PINK1 and the p47 phosphomimic require STIP1 to protect against tau-mediated dendritic loss. Thus, STIP1 is both necessary and sufficient for neuroprotection against tau-mediated dendritic simplification in primary cortical neurons.

## 5. Conclusion

Our study highlights a novel mechanism by which PINK1 protects neurons against tau-induced dendritic simplification, a marker of synaptic loss and neuronal dysfunction in neurodegenerative diseases (Zempel et al., 2017; Ittner and Ittner, 2018; Terry et al., 1991; Lacor et al., 2007). Mutation of either PINK1 or VCP contributes to familial cognitive dysfunction and neurodegeneration (Ricciardi et al., 2014; Piredda et al., 2020; Johnson et al., 2010; Scarian et al., 2022), and their protein-protein interaction promotes phosphorylation of the VCP cofactor p47 to enhance dendritic complexity (Wang et al., 2018). The current data implicate components of the Rho-GTPase interaction pathway downstream of p47 phosphorylation. The molecular chaperone STIP1 is a crucial downstream factor of this neuroprotective signaling pathway, as knockdown of STIP1 blocks the neuroprotective effects of both PINK1 and the p47 phosphomimic. The PINK1-(phospho)p47-STIP1 signaling axis is capable of rescuing dendritic simplification and synaptic loss in a tauopathy model. The data further implicate mitochondrial translation as a potential additional mechanism underlying PINK1-(phospho)p47-mediated neuroprotection, which will be an interesting topic for future studies.

## Supplementary Material

1

2

3

4

5

6

7

8

## Figures and Tables

**Fig. 1.**
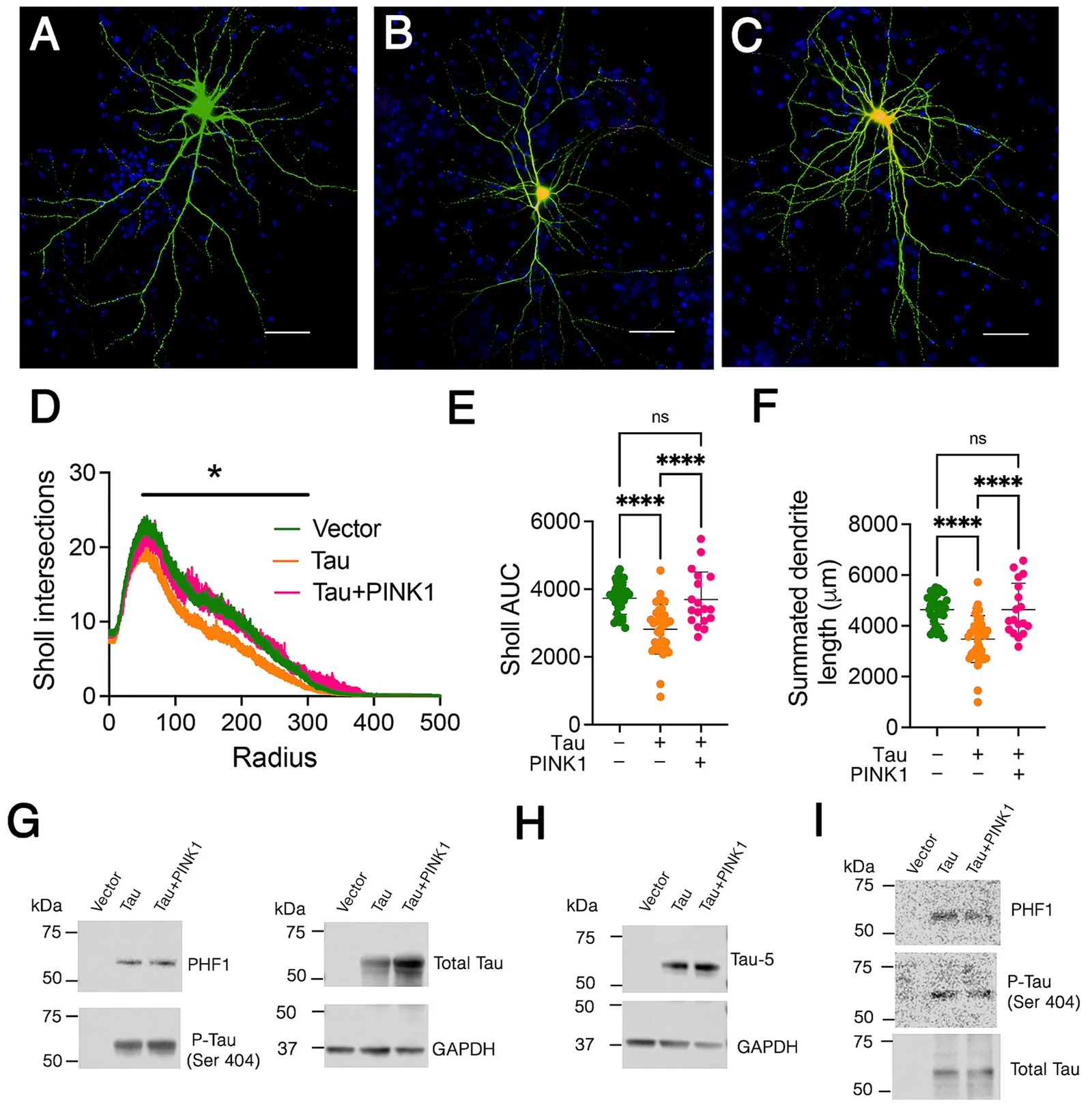
Tau causes dendritic simplification and PINK1 elevation protects against tau-mediated dendrite shortening. Mouse primary cortical neurons transfected with (A) Vector, (B) Tau or (C) Tau+PINK1 were visualized using Venus co-transfection. Neurons were immunostained for His-tagged Tau using an anti-His antibody (red channel). Scale bar = 20 μm. (D) Sholl-curve, (E) Sholl-AUC, and (F) summated dendrite length for each neuron show dendritic simplification resulting from elevated Tau expression, with rescue by PINK1. Panel D: F (2, 81) = 18.25, *p* < 0.0001, 2-way repeated measures ANOVA; * *p* < 0.05, post-hoc Tukey corrected radii. Panel E: F (2, 83) = 19.01, *p* < 0.0001; ****p < 0.0001; ns, non-significant, one-way ANOVA with Tukey’s multiple comparison test. Panel F: F (2, 83) = 19.97, *p* < 0.0001; ****p < 0.0001; ns, non-significant, one-way ANOVA with Tukey’s multiple comparison test. *n* = 32 (Vector), 36 (Tau), 18 (Tau+PINK1) neurons from 5 independent experiments. Representative immunoblots examining the impact of PINK1 elevation on phosphorylated and total tau levels relative to GAPDH are shown for soluble (G, H) and insoluble (I) fractions. See Additional file 1: Fig. S1 for densitometry data normalizing to GAPDH, Tau-5 and Total Tau. See Additional file 3 and 4: Fig. S3 and Fig. S4 for uncropped blots. (For interpretation of the references to colour in this figure legend, the reader is referred to the web version of this article.)

**Fig. 2.**
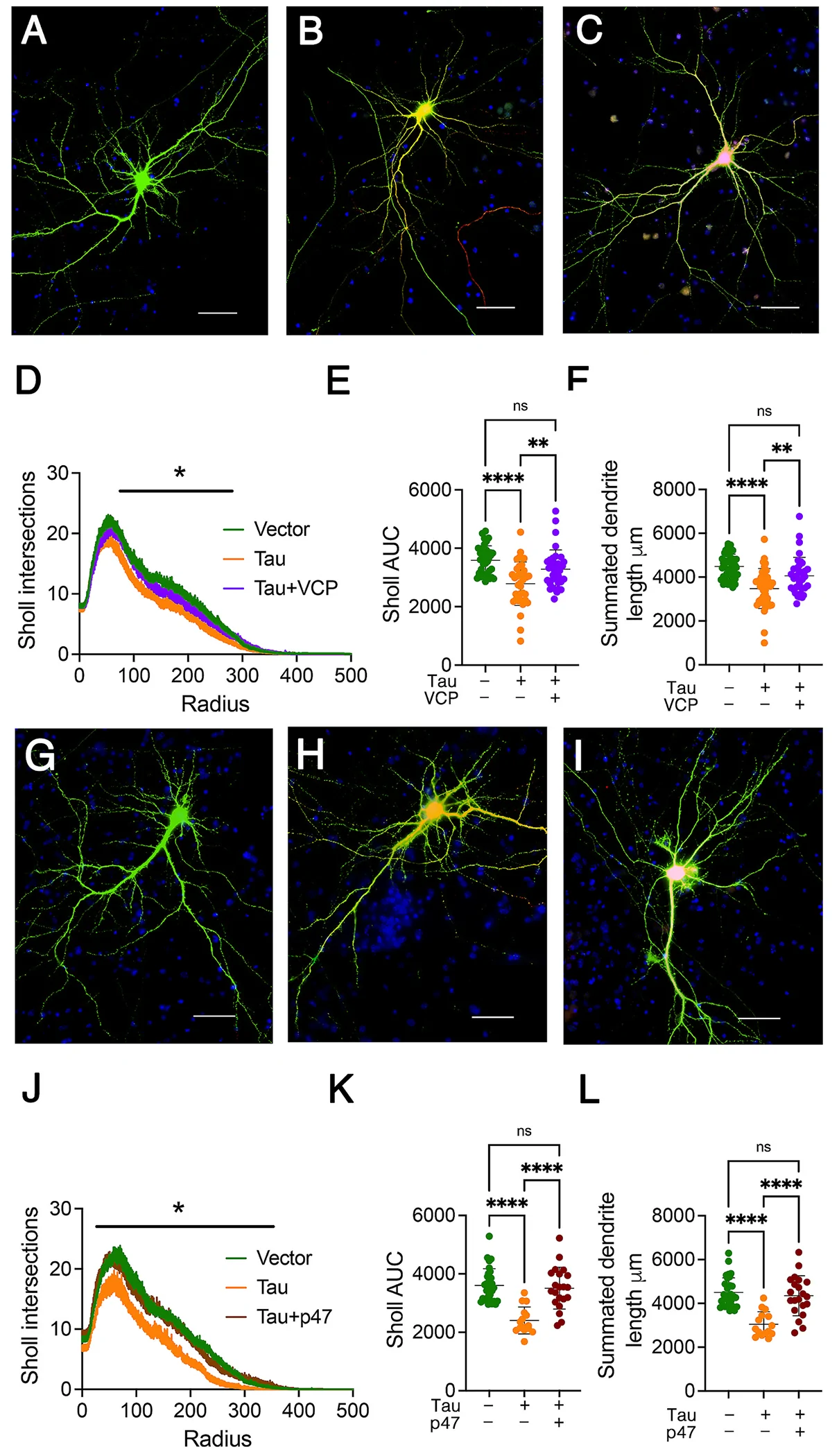
Overexpression of VCP and p47 rescues tau-induced dendritic simplification. Mouse primary cortical neurons transfected with (A) Vector, (B) Tau or (C) Tau+VCP were visualized by Venus co-transfection. Neurons were immunostained for His-tagged Tau using an anti-His antibody (red channel). Scale bar = 20 μm. (D) Sholl-curve, (E) Sholl-AUC, and (F) summated dendrite length for each neuron show rescue by VCP. Panel D: F (3, 114) = 9.926, *p* < 0.0001, 2-way repeated measures ANOVA; *Tukey corrected radii with *p* < 0.05. Panel E: F (2, 109) = 15.28, *p* < 0.0001; ****p < 0.0001; **p = 0.0039; ns, non-significant, one-way ANOVA with Tukey’s multiple comparison test. Panel F: F (2, 109) = 15.83, *p* < 0.0001; ****p < 0.0001; **p = 0.0062; ns, non-significant, one-way ANOVA with Tukey’s multiple comparison test. *n* = 40 (Vector), 37 (Tau), 35 (Tau+VCP) neurons from 6 independent experiments. Mouse primary cortical neurons transfected with (G) Vector, (H) Tau or (I) Tau+p47 were visualized by Venus co-transfection and immunostained for His-tagged Tau using an anti-His antibody (red channel). Scale bar = 20 μm. (J) Sholl-curve, (K) Sholl-AUC, and (L) summated dendrite length for each neuron show rescue by p47. Panel J: F (2, 63) = 21.55, *p* < 0.0001, 2-way repeated measures ANOVA; **p < 0.05, Tukey corrected radii. Panel K: F (2, 63) = 21.55, *p* < 0.0001; ****p < 0.0001; ns, non-significant, one-way ANOVA with Tukey’s multiple comparison test. Panel L: F (2, 64) = 21.16, *p* < 0.0001; ****p < 0.0001; ns, non-significant, one-way ANOVA with Tukey’s multiple comparison test. *n* = 30 (Vector), 15 (Tau), 21 (Tau+p47) neurons from 5 independent experiments. (For interpretation of the references to colour in this figure legend, the reader is referred to the web version of this article.)

**Fig. 3.**
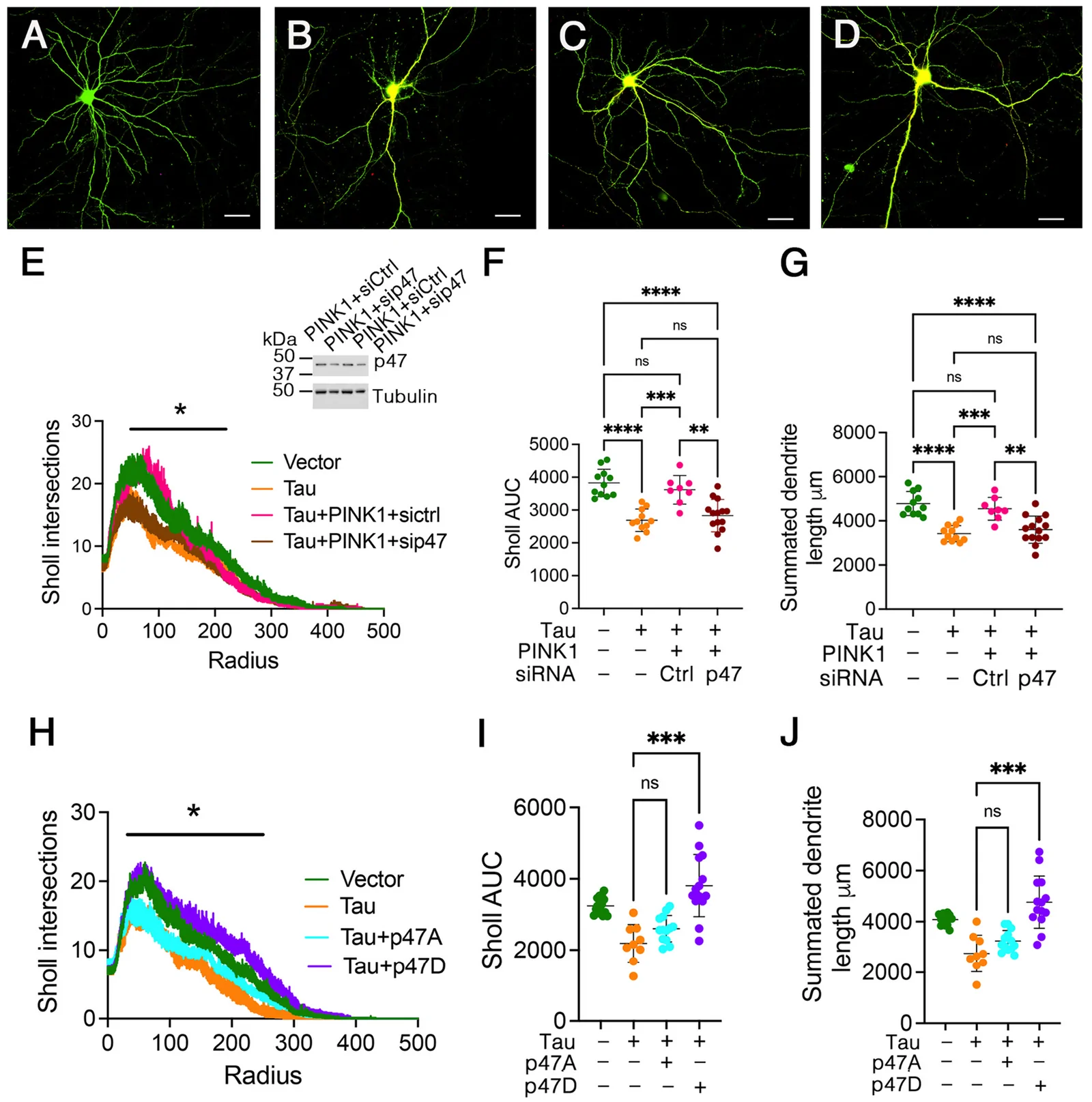
PINK1 requires p47 to protect against Tau and the p47 phosphorylation mimic rescues dendritic complexity. Mouse primary cortical neurons transfected with (A) Vector, (B) Tau, (C) Tau+PINK1 + sictrl or D) Tau+PINK1 + sip47 were visualized by Venus co-transfection, and immunostained for His-tagged Tau using an anti-His antibody (red channel). Scale bar = 20 μm. (E) Sholl-curve, (F) Sholl-AUC, and (G) summated dendrite length for each neuron show that sip47 inhibits the ability of PINK1 to protect against tau. Inset E: sip47 knockdown in primary neurons (See Additional file 5: Fig. S5 A–B for uncropped blots). Panel E: F (3, 40) = 18.84, *p* < 0.0001, 2-way repeated measures ANOVA; *Tukey corrected radii with *p* < 0.05. Panel F: F (3,40) = 18.84, *p* < 0.0001; ****p < 0.0001; ***p = 0.0002; **p = 0.0010; ns, non-significant, one-way ANOVA with Tukey’s multiple comparison test. Panel G: F (3, 40) = 18.15, *p* < 0.0001; ****p < 0.0001; ***p = 0.0002; **p = 0.0012; ns, non-significant, one-way ANOVA with Tukey’s multiple comparison test. *n* = 11 (Vector), 11 (Tau), 8 (Tau+PINK1 + sictrl), 14 (Tau+PINK1 + sip47) neurons compiled from 2 independent experiments. Cortical neurons were transfected with Vector, Tau, Tau+p47A or Tau+p47D, and visualized using Venus co-transfection. (H) Sholl-curve, (I) Sholl-AUC, and (J) summated dendrite length for each neuron show rescue by p47D of each parameter of tau-induced dendritic simplification. Panel H: F (3, 47) = 19.21, *p* < 0.0001, 2-way repeated measures ANOVA; *Tukey corrected radii with *p* < 0.05. Panel I: F (3.000, 27.41) = 19.31, *p* < 0.0001; ***p = 0.0001; ns, non-significant, Brown-Forsythe ANOVA with Dunnett’s T3 multiple comparisons test. Panel J: F (3.000, 26.45) = 20.85, *p* < 0.0001; ***p = 0.0001; ns, non-significant, Brown-Forsythe ANOVA with Dunnett’s T3 multiple comparisons test. *n* = 15 (Vector), 9 (Tau), 13 (Tau+p47A), 14 (Tau+p47D) neurons from 3 independent experiments. (For interpretation of the references to colour in this figure legend, the reader is referred to the web version of this article.)

**Fig. 4.**
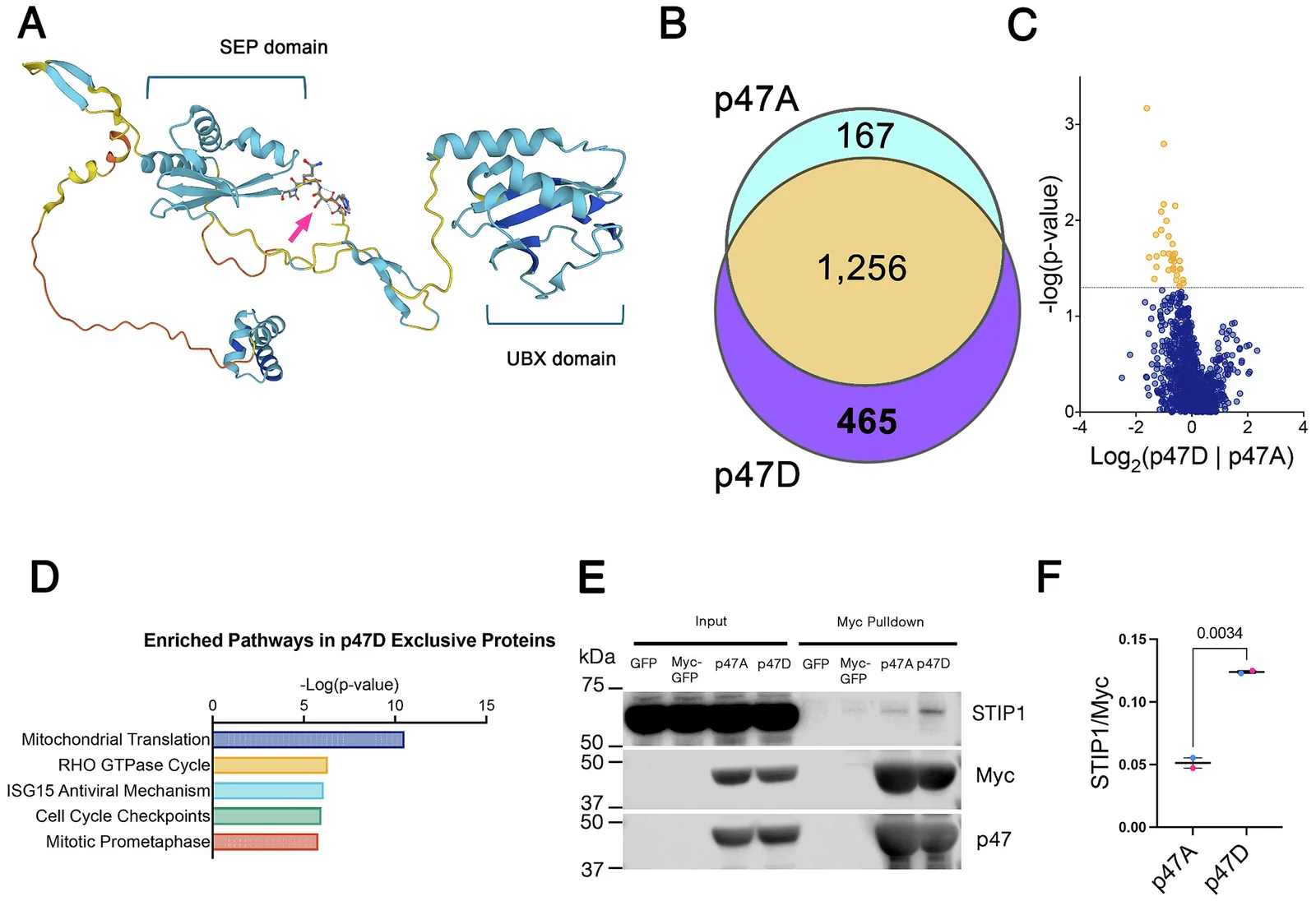
IP-MS reveals differentially enriched proteins for p47D. (A) Alpha-fold structure (Varadi et al., 2022; Jumper et al., 2021) of p47 showing the location of residue S176 (pink arrow) in relation to the UBX domain, which mediates p47 interactions with VCP (Dreveny et al., 2004), and the SEP domain, which regulates p47 trimerization and VCP interactions (Yuan et al., 2004). (B) IP-MS performed on three independent biological samples per group revealed 1888 proteins identified in the p47 pulldowns after subtracting peaks present in the GFP-transfected control. The distribution of proteins identified in p47A and p47D pulldowns is shown in the Venn diagram. (C) Volcano plot of 1256 proteins pulled down by both p47A and p47D reveals 34 proteins (in yellow) nominally enriched in the p47A pulldowns. Pathway analysis for these proteins and for the 167 proteins exclusive to the p47A pulldowns are shown in Additional file 2: Fig. S2. (D) Pathway analysis of the 465 proteins exclusive to the p47D pulldowns. To validate differential binding of STIP1, HEK293 cells transfected with Myc-GFP (negative control), Myc-p47A, or Myc-p47D were lysed and immunoprecipitated using anti-Myc antibodies. Immunoblot (E) and densitometry (F) data show that p47D pulls down significantly more STIP1 than p47A. (*p* = 0.0034, unpaired *t*-test, *N* = 2 independent experiments; see Additional file 6: Fig. S6 A–B for uncropped blots). (For interpretation of the references to colour in this figure legend, the reader is referred to the web version of this article.)

**Fig. 5.**
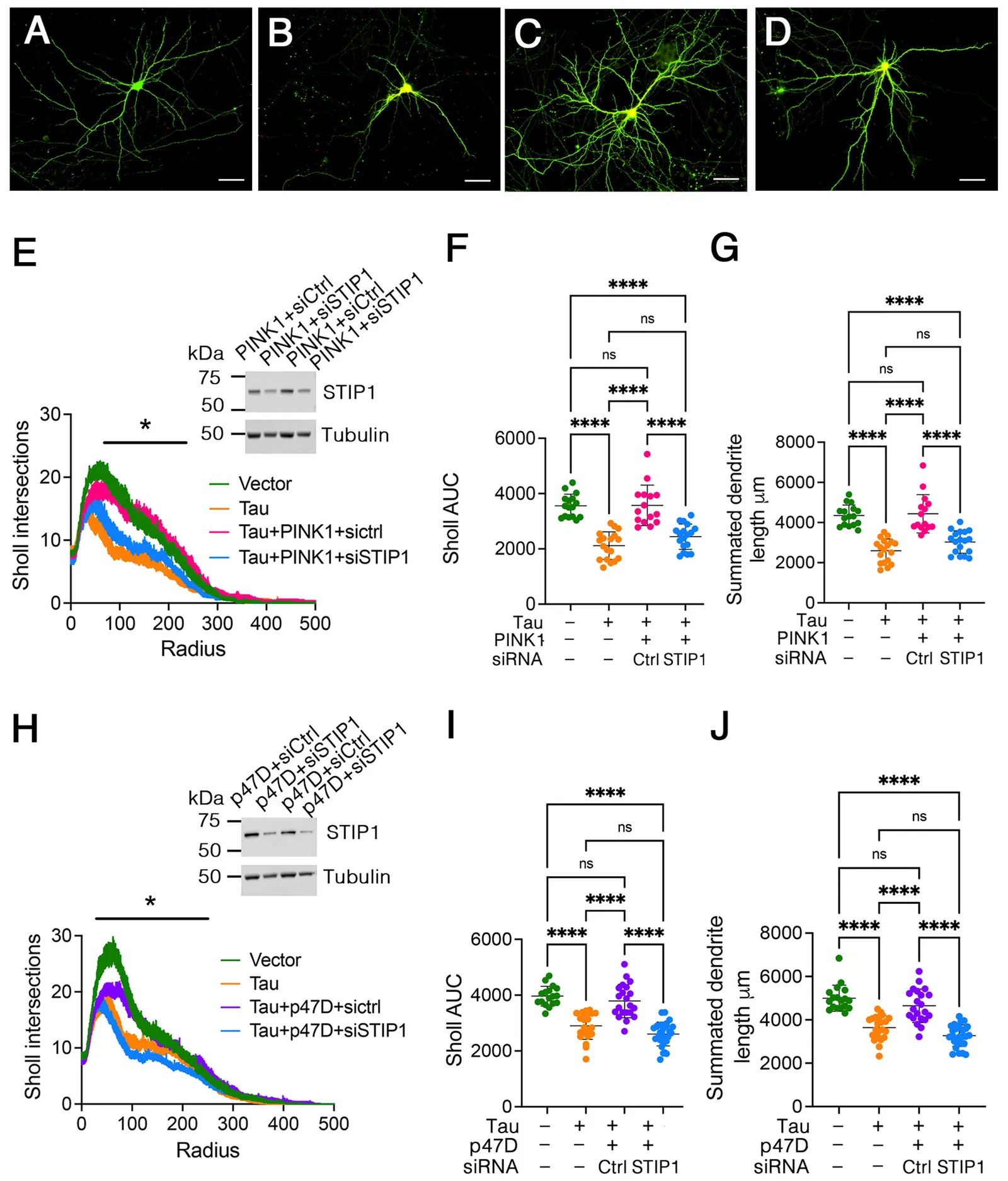
PINK1 and p47D require STIP1 to protect against tau-induced dendritic simplification. Mouse primary cortical neurons transfected with (A) Vector, (B) Tau, (C) Tau+PINK1 + sictrl or D) Tau+PINK1 + siSTIP1 were visualized by Venus co-transfection. Neurons were immunostained for His-tagged Tau using an anti-His antibody (red channel). Scale bar = 20 μm. (E) Sholl-curve, (F) Sholl-AUC, and (G) summated dendrite length for each neuron show knockdown of STIP1 blocks PINK1-mediated protection against tau-induced dendritic simplification. Inset: siSTIP1 knockdown in primary neurons (See Additional file 5: Fig. S5 E–F for uncropped blots). Panel E: F (3, 63) = 33.45, *p* < 0001, 2-way repeated measures ANOVA; *Tukey corrected radii with *p* < 0.05; Panel F: F (3, 63) = 33.58, *p* < 0.0001; ****p < 0.0001; ns, non-significant, one-way ANOVA with Tukey’s multiple comparison test; Panel G: F (3, 63) = 32.51, *p* < 0.0001; ****p < 0.0001; ns, non-significant, one-way ANOVA with Tukey’s multiple comparison test. *n* = 16 (Vector), 18 (Tau), 15 (Tau+PINK1 + sictrl), 18 (Tau+PINK1 + siSTIP1) neurons from 4 independent experiments. Primary cortical neurons were transfected with Vector, Tau, Tau+p47D + sictrl or Tau+p47D + siSTIP1, and visualized using Venus co-transfection. (H) Sholl-curve, (I) Sholl-AUC, and (J) summated dendrite length for each neuron show that knockdown of STIP1 blocks p47D-mediated protection against tau-induced dendritic simplification. Inset: siSTIP1 knockdown in primary neurons (See Additional file 5: Fig. S5 C–D for uncropped blots). Panel H: F (3, 85) = 39.62, *p* < 0.0001, 2-way repeated measures ANOVA; *Tukey corrected radii with *p* < 0.05. Panel I: F (3, 84) = 42.71, *p* < 0.0001; ****p < 0.0001; ns, non-significant, one-way ANOVA with Tukey’s multiple comparison test. Panel J: F (3, 85) = 40.51, *p* < 0.0001; ****p < 0.0001; ns, non-significant, one-way ANOVA with Tukey’s multiple comparison test. *n* = 17 (Vector), 22 (Tau), 21 (Tau+p47D + sictrl), 28 (Tau+p47D + siSTIP1) neurons from 3 independent experiments. (For interpretation of the references to colour in this figure legend, the reader is referred to the web version of this article.)

**Fig. 6.**
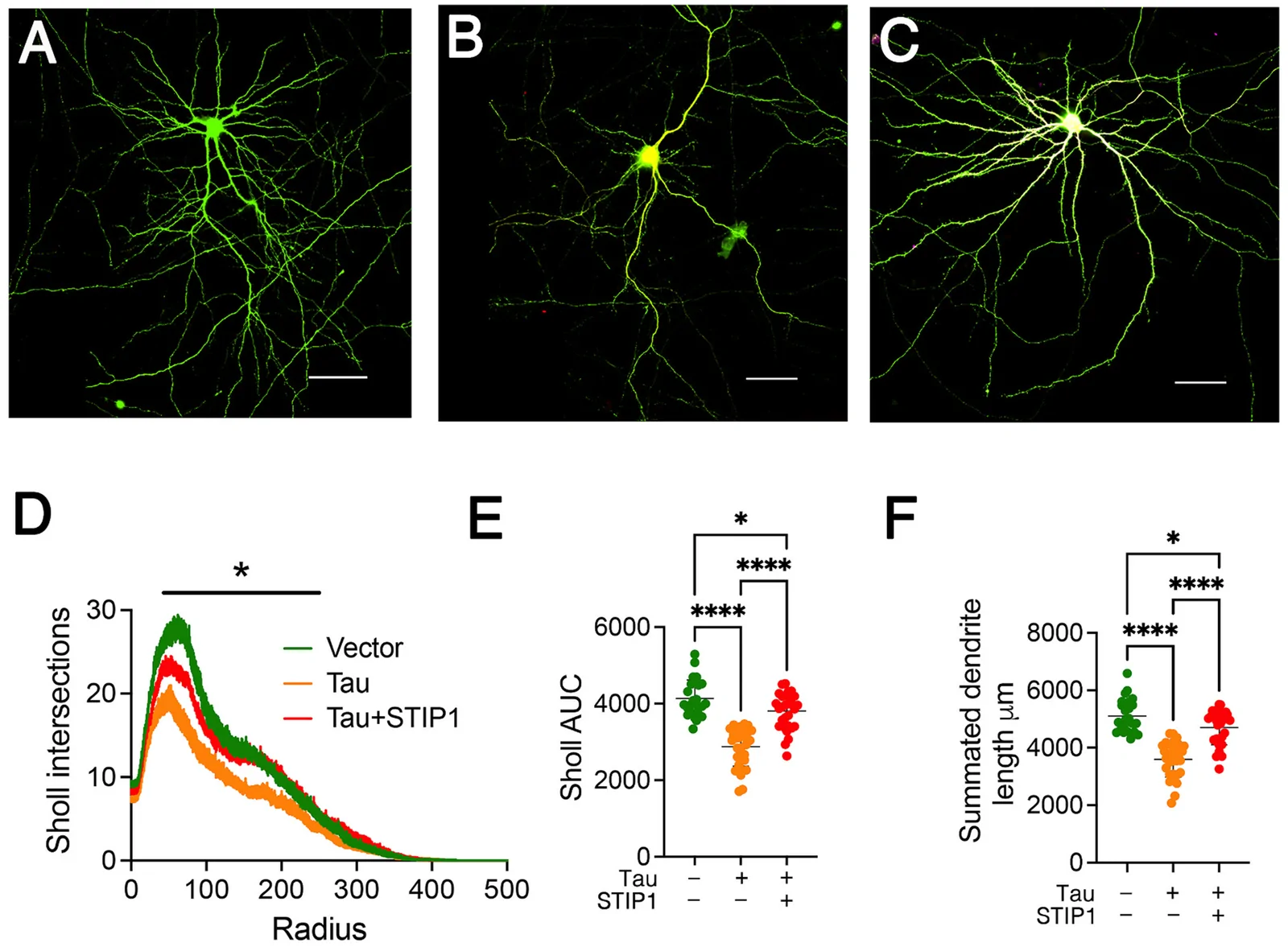
STIP1 elevation protects against tau. Mouse primary cortical neurons transfected with (A) Vector, (B) Tau or (C) Tau+STIP1 were visualized by Venus co-transfection, and immunostained for His-tagged Tau (red channel). Scale bar = 20 μm. (D) Sholl-curve, (E) Sholl-AUC, and (F) summated dendrite length for each neuron show significant rescue by STIP1. Panel D: F (2, 83) = 51.09, *p* < 0.0001, 2-way repeated measures ANOVA; *Tukey corrected radii with *p* < 0.05. Panel E: F (2, 83) = 51.09, *p* < 0.0001; ****p < 0.0001; *p = 0.0400, one-way ANOVA with Tukey’s multiple comparison test. Panel F: F (2, 83) = 48.81, *p* < 0.0001; ****p < 0.0001; *p = 0.0419, one-way ANOVA with Tukey’s multiple comparison test. *n* = 25 (Vector), 31 (Tau), 30 (Tau+STIP1) neurons from 3 independent experiments. (For interpretation of the references to colour in this figure legend, the reader is referred to the web version of this article.)

**Table 1:** Proteins of the RHO GTPase Cycle Pathway that associated with p47D. Spectral quantification for each sample was normalized to its respective p47 abundance, and the mean for each protein ranked by relative abundance. The STIP1 antibody detected an appropriately sized band and subsequent co-immunoprecipitation validated its interaction with p47D.

Symbol	Entrez gene name	UniProt ID
SPTAN1	spectrin alpha, non-erythrocytic 1	Q13813
DOCK7	dedicator of cytokinesis 7	Q96N67
SCRIB	scribble planar cell polarity protein	Q14160
SHMT2	serine hydroxymethyltransferase 2	P34897
IQGAP3	IQ motif containing GTPase activating protein 3	Q86VI3
**STIP1**	**stress induced phosphoprotein 1**	**P31948**
MYO6	myosin VI	Q9UM54
UACA	uveal autoantigen with coiled-coil domains and ankyrin repeats	Q9BZF9
ECT2	epithelial cell transforming 2	Q9H8V3
GNA13	G protein subunit alpha 13	Q14344
SCFD1	sec1 family domain containing 1	Q8WVM8
GPS1	G protein pathway suppressor 1	Q13098
TXNL1	thioredoxin like 1	O43396
ARHGEF18	RhoRac guanine nucleotide exchange factor 18	Q6ZSZ5
CSK	C-terminal Src kinase	P41240
RBBP6	RB binding protein 6, ubiquitin ligase	Q7Z6E9
ARHGDIA	Rho GDP dissociation inhibitor alpha	P52565
MTX1	metaxin 1	Q13505
PAK4	p21 (RAC1) activated kinase 4	O96013
NDUFA5	NADH:ubiquinone oxidoreductase subunit A5	Q16718
ABI2	abl interactor 2	Q9NYB9
LMAN1	lectin, mannose binding 1	P49257
WDR11	WD repeat domain 11	Q9BZH6
CDC37	cell division cycle 37, HSP90 cochaperone	Q16543
SRGAP2	SLIT-ROBO Rho GTPase activating protein 2	Q91Z67
VAPB	VAMP associated protein B and C	O95292

## Data Availability

The mass spectrometry data have been deposited to the ProteomeXchange Consortium (http://proteomecentral.proteomexchange.org) *via* the MassIVE partner repository with the dataset identifier MSV000098502 (https://massive.ucsd.edu). The other data supporting the findings of this study are included within the article and its additional files.

## Appendix A. Supplementary data

Supplementary data to this article can be found online at https://doi.org/10.1016/j.nbd.2026.107592.
