# Supplementary material for: The PINK1-VCP-p47 pathway protects against tau-induced dendritic shrinkage in cortical neurons through interaction with STIP1
Source: Neurobiol Dis. Author manuscript; Available in PMC 2026 Oct 16. (PMC13674045; doi:10.1016/j.nbd.2026.107592)
Supplement: Supplementary file 1 [file NIHMS2214720-supplement-1.pdf]

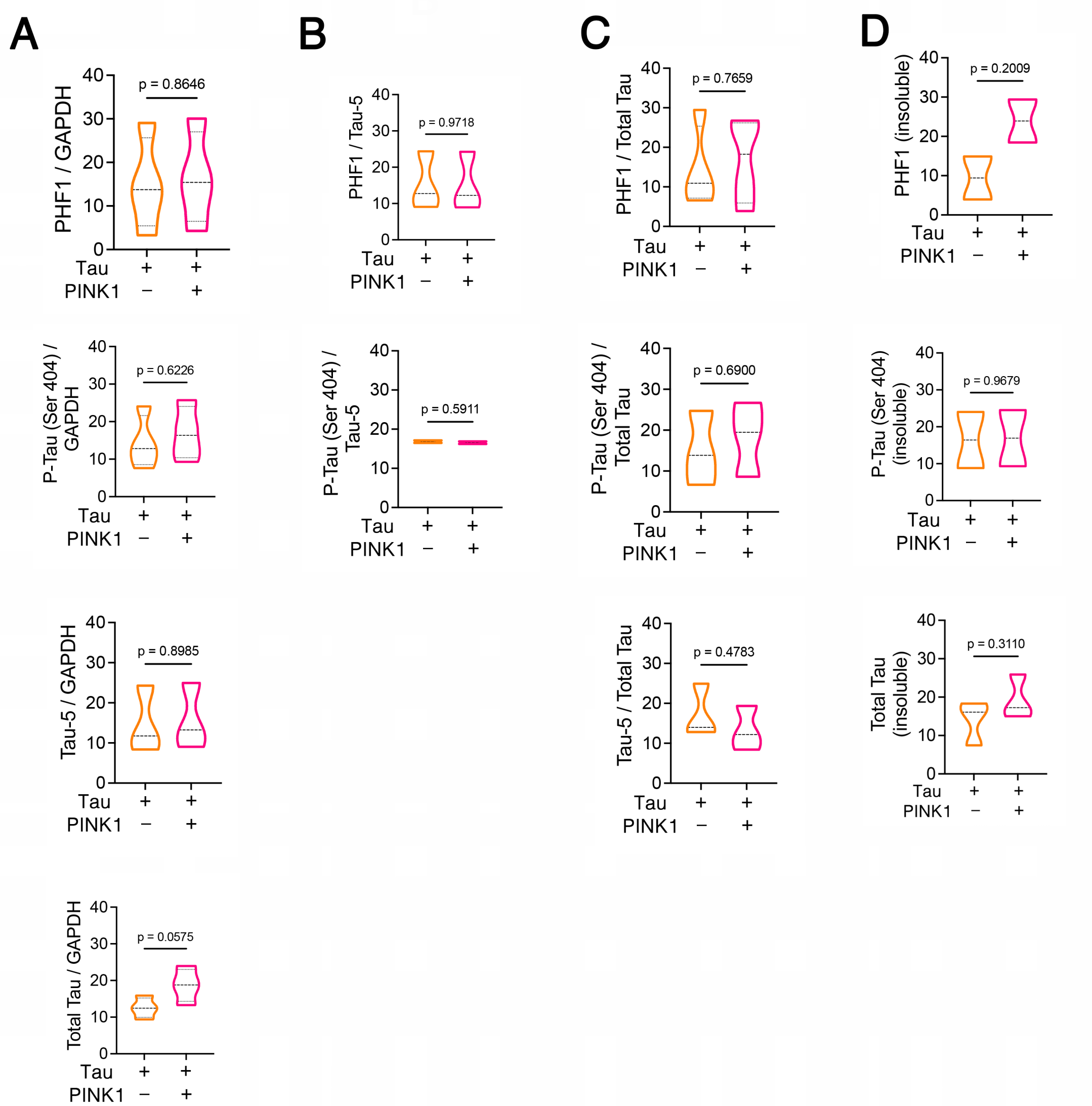

Additional file 1: Figure S1: PINK1 elevation does not affect tau expression (related to Figure 1 G-I). Densitometry accompanying representative soluble immunoblots shown in Figure 1. Panel A-C represent soluble fraction quantification normalized to GAPDH, Tau-5 and total Tau respectively. Panel D represents insoluble fraction quantification normalized to soluble GAPDH. p values from unpaired Student's t-test are shown.
N=4: PHF1/GAPDH, P-Tau (Ser 404)/GAPDH, Total Tau/GAPDH, PHF1/Total Tau);
N=3: Tau-5/GAPDH, P-Tau/Total Tau, Tau-5/Total Tau, PHF1/Tau-5, Total tau insoluble;
N=2: P-Tau (Ser 404)/Tau-5, PHF1 insoluble and P-Tau insoluble.
